# Salt Restriction and Angiotensin-Converting Enzyme Inhibitor Improve the Responsiveness of the Small Artery in Salt-Sensitive Hypertension

**DOI:** 10.7150/ijms.79741

**Published:** 2023-03-05

**Authors:** Shi-Cheng Li, Tong-Meng Jiang, Jia-Hao Zhang, Meng-Ying Zeng, Yu-Xin Ma, Shu-Yi Feng, Qing-Hai Wang, Xiao-Wei Yan

**Affiliations:** 1 Department of Cardiology, Peking Union Medical College Hospital (PUMCH), Chinese Academy of Medical Sciences & Peking Union Medical College, Beijing 100730, China.; 2 Laboratory of Hainan Trauma and Disaster Rescue, The First Affiliated Hospital of Hainan Medical University, Haikou 571199, China.; 3 Key Laboratory of Emergency and Trauma, Ministry of Education, Engineering Research Center for Hainan Bio-Smart Materials and Bio-Medical Devices, Key Laboratory of Hainan Functional Materials and Molecular Imaging, College of Emergency and Trauma, Hainan Medical University, Haikou 571199, China.; 4 Experimental Research Center, China Academy of Chinese Medical Sciences, Beijing 100700, China.; 5 Department of Cardiology, the Second Hospital, Cheeloo College of Medicine, Shandong University, Jinan 250000, China.

**Keywords:** Hypertension, salt intake, salt restriction, mesenteric small artery, vascular reactivity, angiotensin-converting enzyme (ACE) inhibitor

## Abstract

For salt-sensitive hypertension (SSH), salt restriction and angiotensin-converting enzyme (ACE) inhibitors are essential treatments, but their effect on the function of resistance arteries is unclear. Here, we present an intravital study to detect the effect of salt restriction and ACE inhibitors on the function of the mesenteric small artery (MSA) in SSH. Dahl salt-sensitive rats were randomized into the following groups: ACE inhibitor gavage, salt restriction, ACE inhibitor combined with salt restriction, and high-salt diet. After a 12-week intervention, the mesenteric vessels maintained their perfusion *in vivo*, and the changes in the diameter and blood perfusion of the MSAs to norepinephrine (NE) and acetylcholine (ACh) were detected. Switching from a high-salt diet to a low-salt diet (i.e., salt restriction) attenuated the vasoconstriction of the MSAs to NE and promoted the vasodilatation to ACh, while ACE inhibitor improved the vasodilatation more obviously. Pathologically, changes in local ACE, AT1R, and eNOS expression were involved in these processes induced by a high-salt diet. Our study suggests that salt restriction and ACE inhibitor treatment improve high salt intake-induced MSA dysfunction in SSH, and salt restriction is a feasible and effective treatment. Our findings may provide a scientific basis for the treatment of hypertension.

## Introduction

More than 31.1% of the global adult population worldwide were affected by hypertension 1, and approximately 50-60% of hypertensives are salt sensitive 2. Increased salt consumption is associated with increased BP and cardiovascular diseases 3, far more than < 5 grams per day, the WHO-recommended amount 4. In addition, increased salt sensitivity usually occurs in obesity, metabolic syndrome, and aging people despite genetic polymorphisms 2, 5. Therefore, the high salt intake of hypertensive individuals has become a problem worthy of attention and urgently needs to be solved. Although a low-salt diet is beneficial to improve the retention of water and sodium in hypertensive individuals, its effect on the function of resistance arteries and the cooperative effect with antihypertensive drugs have not been fully demonstrated.

In addition to regulating blood flow to organs, small resistance arteries contribute to total peripheral vascular resistance, which is a crucial factor affecting blood pressure 6. The increases 7, 8, decreases 9, 10 and biphasic 11, 12 effects of the vascular response induced by vasoconstrictors and vasodilators in arterial tonus have been reported previously. In a study of salt and vasomotor function, a high-salt diet reduced endothelium-dependent relaxation of the thoracic aorta in response to ACh *in vitro* 13, and high salt intake induced increased alpha-1 receptor responsiveness in excised MSAs 14. Angiotensin-angiotensin system (RAS) is one of the mechanisms that facilitate arterial perfusion 15, because it cannot be completely simulated by isolated blood vessels with Krebs buffer or saline solution 11, especially vascular reactions to various agents. In addition, in real life, excessive salt intake is a common problem in hypertensive individuals. High salt intake negatively influenced the response of resistant arteries to vasoactive agents, as confirmed by our previous study 16. Therefore, we hope to further explore the relationship between resistance artery and BP from another perspective by limiting salt intake.

Currently, there is a global consensus to limit salt intake; in particular, the benefits of changing from a high to a low salt intake beyond lower BP are noteworthy. Therefore, in the present study, we assessed the impact of changing from high salt intake to low salt intake on the vascular reactivity of mesenteric small arteries (MSA) *in vivo* on the basis of benazepril, an angiotensin-converting enzyme (ACE) inhibitor, in the treatment of salt-sensitive hypertension.

## Materials and Methods

### Experimental animals

In all experiments, the US National Institutes of Health's Guide for the Care and Use of Laboratory Animals 17 and ARRIVE guidelines were followed 18. Ethical approval for this study was granted by the Ethical Committee of PUMCH (XHDW-2017-011).

All male Dahl-SS rats were obtained from Vital River Laboratory Animal Technology Co. Ltd. (Beijing, China), weighed 200-230 grams, and were maintained on a 12-h light/dark cycle with unrestricted access to food and drinking water. After 7 days of adaptation, rats were randomly divided into 4 experimental groups and the feed was changed at the same time: HS (high-salt diet, 8% NaCl content feed^19^, n = 8) for 12 weeks, HB (HS with Benazepril administered intragastrically, n = 9) for 12 weeks, HLS (high salt for the first 6 weeks, then low-salt diet for the next 6 weeks, 0.3% NaCl content feed, n = 8), and HLB (HLS with Benazepril administration, 10 mg/kg per day, n = 9) for 12 weeks. Feed quantities of 8% HS and 0.3% LS were adjusted according to Beijing Keao Xieli Feed CO., LTD. normal feed. A tail-cuff method (BP-2010A, Softron) was used to monitor blood pressure before randomization and at 6 and 12 weeks.

### Biochemical analysis and vascular preparation

The inner canthal orbital vein blood and 24-hour urine samples were collected at the end of the 12^th^ week, and analyzed by the Clinical Laboratory of PUMCH (Olympus AU5400 and SIEMENS Clinitek Atlas Analyzer), including for serum creatinine, potassium, 24 hours urinary sodium and urinary total protein. The plasma angiotensin II and aldosterone levels were measured by radioimmunoassay. The proximal small intestine and mesentery were removed and placed in a transparent chamber filled with a constant temperature physiological saline solution after pentobarbital sodium (30 mg/kg, Sigma-Aldrich) was administered intraperitoneally 16. The mesenteric vessels maintained their perfusion and vascular tone *in vivo* and *in situ*.

### MSA diameter changes in response to vasoactive agents

To evaluate the changes in the MSAs* in vivo*, the start time, a GigaView Suite was used to record the maximum changes, the time to maximum changes and the duration of vasoconstriction/vasodilation in the second branch of MSAs induced by vasoactive agents. These results for each rat were averaged from three segments of the MSAs. The optimal reaction concentrations 16 of both norepinephrine tartrate (NE) and acetylcholine chloride (ACh) were 10 μg/kg. The dynamic change in the MSA diameter was recorded immediately after NE and ACh injection, and the interval between injections was at least 15 minutes to ensure drug elution. The GigaView Suite includes a microscope (BX51W1, OLYMPUS), high-speed camera (GigaView, Southern Vision Systems Inc.) and supporting software (GigaView software, version 2.4.7). Image-Pro Plus 6.0 software was used to determine MSAs diameters. The MSA under a microscope are shown in Figure S1 and Video S1.

### Measuring the effect of drug injection on MSA perfusion

To evaluate the changes in MSA blood perfusion *in vivo*, a full-field laser perfusion imaging system (LPI) (Moor Instruments Ltd., UK) was used to continuously record and analyze the small arteries of the proximal small intestine 20. According to the measurement unit of the LPI suite software, blood perfusion was expressed as the perfusion unit (PU). An average blood perfusion of MSAs was determined between 10th and 40th seconds after NE or ACh injection, based on the baseline blood perfusion. In the 30 s interval, the maximum MSA perfusion changes relative to baseline were recorded. For each rat, the blood perfusion of three arteries was averaged. Analyzing the LPI measurements was carried out using moorFLPI reviewer version 3.0 (Moor Instruments Ltd.). The MSAs under LPI are shown in Figure S1.

### Histological analysis

After measurement *in vivo*, the MSAs of each rat were fixed, embedded and sliced. Hematoxylin and eosin, Masson staining and immunohistochemistry were performed on the MSAs in sections. Immunohistochemistry detecting the expression of angiotensin II type 2 receptor (AT2R), angiotensin II type 1 receptor (AT1R), angiotensin-converting enzyme (ACE) and endothelial nitric oxide synthase (eNOS). All these antibodies were purchased from Abcam. To determine the rate of fibrosis or protein expression, Image-Pro Plus 6.0 (Media Cybemetics, USA) was used to calculate the ratio of positive expression areas in the media or endothelium.

### Statistical analysis

The means and standard deviations of the data are given. Students' t-tests or one-way ANOVA were used to compare groups. GraphPad Prism 8.0.2 was used for all analyses and a significance level of p < 0.05 was used.

## Results

An anesthetic accident during surgery caused the death of two rats in the HS group and two rats in the HLS group after a 12-week dietary intervention. The dead rats completed all tests except the MSAs under a microscope and LPI, and the remaining rats completed all the experimental procedures.

### General characteristics

**Figure 1:**
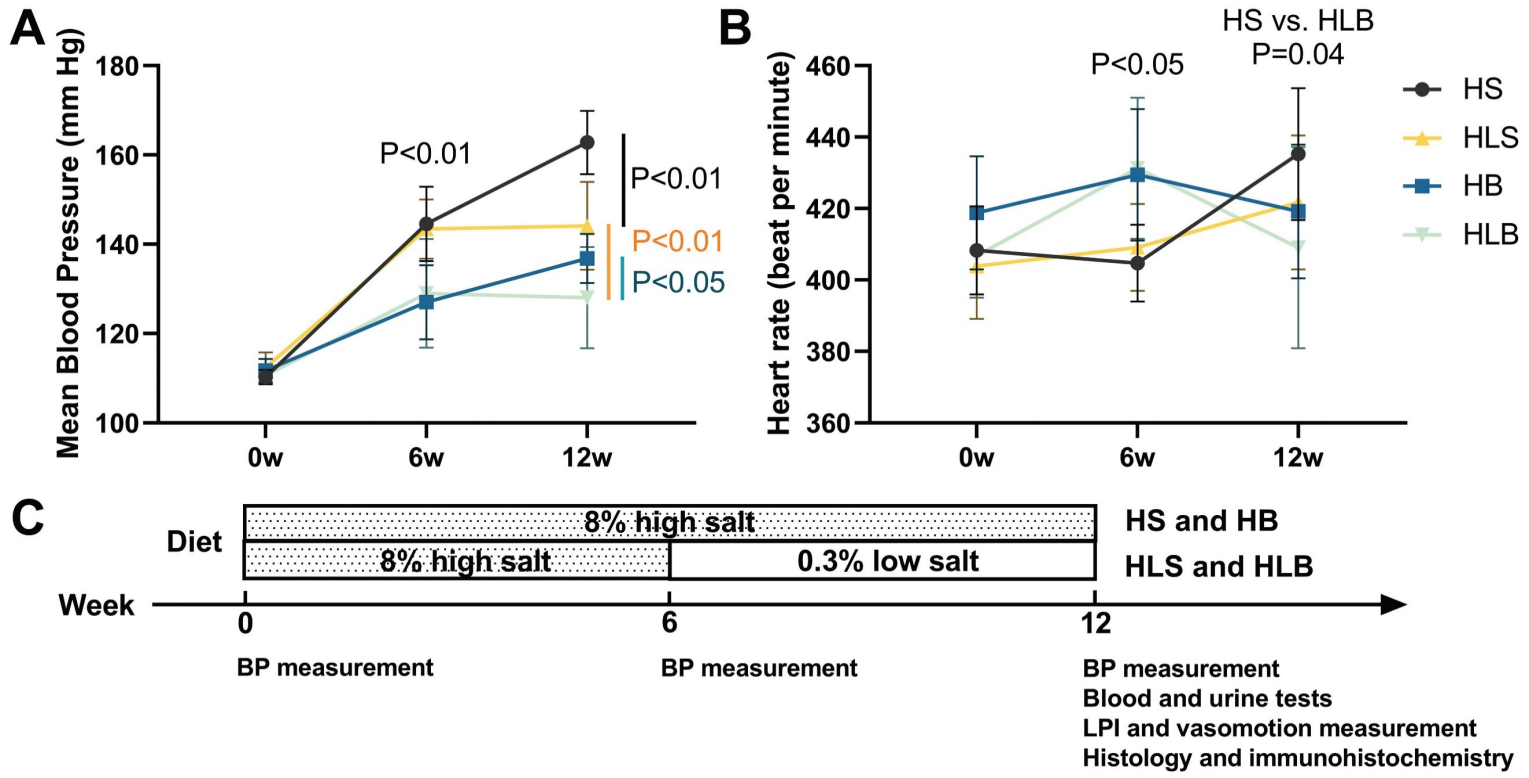
The mean blood pressure (A) and heart rate (B) of Dahl-SS rats at baseline and at the end of the 6-week and 12-week dietary interventions. (C) Timeline of the protocol. HS: the group with high salt intake for 12 weeks, n=8; HLS: the group with high salt for the first 6 weeks, then low salt intake for the next 6 weeks, n=8; HB: the group with high salt intake plus Benazepril administered intragastrically for 12 weeks, n=9; HLB: the group with high salt for the first 6 weeks, then low salt intake for the next 6 weeks, and with Benazepril administration throughout 12 weeks, n=9.

At baseline, neither group's mean blood pressure nor heart rate was significantly different. After 6 weeks of dietary and ACE inhibitor intervention, the mean BP of the high salt with Benazepril (HB, HLB) group was significantly lower than that of the HS without Benazepril (HS, HLS) group (HB vs. HS: P = 0.0006; HLB vs. HLS: P = 0.0093), and the heart rate of the HB and HLB groups was significantly higher than that of the HS and HLS groups (HB vs. HS: P = 0.0046; HLB vs. HLS: P = 0.0152). A significantly lower mean blood pressure was observed in the HB and HLS groups than HS group (P < 0.0001 and P = 0.0007, respectively) after the 12th week, but a significantly higher one than the HLB group (P = 0.0330 and P = 0.0072, respectively). The systolic and mean BP were not significantly different between the HB and HLS groups, but the diastolic BP of the HLS group was higher than that of the HB group (P = 0.0383). Table S1 and Figure 1 show that no difference in heart rate was observed among the four groups after the intervention of 12 weeks (all P > 0.05).

### Biochemical analysis

After 12 weeks of intervention, high salt intake significantly inhibited plasma aldosterone and angiotensin II levels. As expected, low-salt diets significantly reduced 24-hour urinary sodium excretion in both HLS and HLB groups when salt consumption changed from high to low. The 24-hour urinary total protein excretion of the HB and HLS groups was significantly lower than that of the HS group (P = 0.0076 and P = 0.0396, respectively) (Table 1).

### The effects of NE and ACh on MSAs

**Figure 2:**
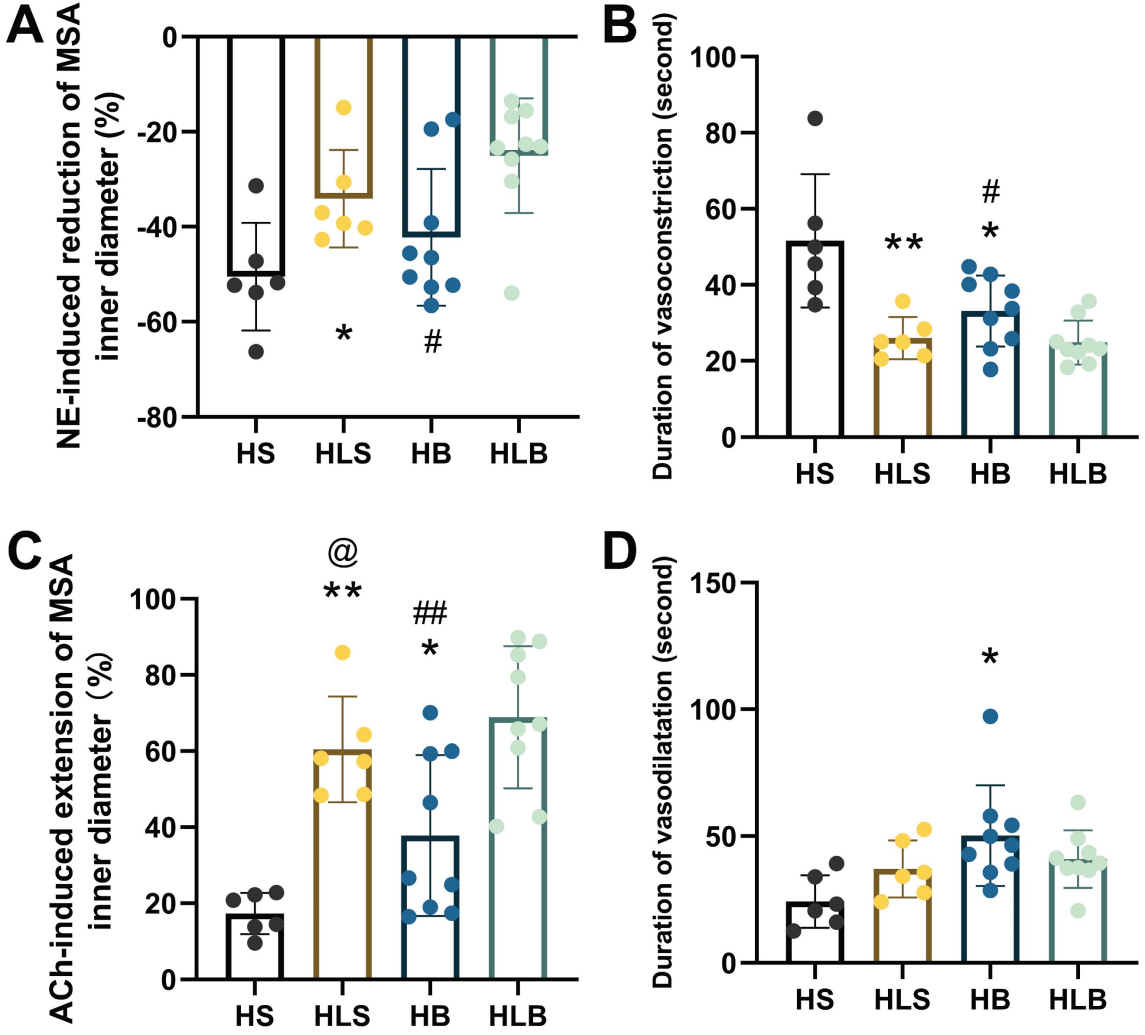
NE-induced vasoconstriction and ACh-induced vasodilatation of MSAs. (A) NE-induced maximum percentage change of the MSA inner vasoconstriction. (B) Duration of NE-induced vasoconstriction, from the beginning of vasoconstriction to the end of artery diameter recovery. (C) ACh-induced maximum percentage change in MSA inner vasodilatation. (D) Duration of ACh-induced vasodilatation, from the beginning of ACh injection to the end of artery diameter recovery. The percentage change in vasoconstriction/vasodilatation was calculated as the percentage of MSA inner diameter changes after NE/ACh injection divided by the baseline inner diameter. NE and ACh were injected through a femoral vein (10 μg/kg). The second order branch of the mesenteric arteries was recorded by a high-speed camera attached to a microscope. The measurements of three MSA segments were averaged. HS: the group with high salt intake for 12 weeks, n=8; HLS: the group with high salt for the first 6 weeks, then low salt intake for the next 6 weeks, n=8; HB: the group with high salt intake plus Benazepril administered intragastrically for 12 weeks, n=9; HLB: the group with high salt for the first 6 weeks, then low salt intake for the next 6-week, and with Benazepril administration throughout 12 weeks, n=9. MSA: mesenteric small artery; NE: norepinephrine; ACh: acetylcholine. *: compared with HS group, P<0.05; **: compared with the HS group, P<0.01; #: compared with the HLB group, P<0.05; ##: compared with the HLB group, P<0.01; @: compared with the HB group, P<0.05.

After NE administration, the maximum percentage reduction in the MSA inner diameter was more significant in the HS group than in the HLS group (-50.48 ± 11.34 vs. -34.13 ± 10.27%, and P = 0.0257), was more significant in the HB group than in the HLB group (-42.25 ± 14.39 vs. -25.03 ± 12.08%, P = 0.0143), and was not different between the HB and HLS groups (Figure 2A). As compared to the HB and HLS groups, the HS group had significantly longer vasoconstriction duration (51.6 ± 17.5 vs. 33.1 ± 9.3 and 26.0 ± 5.5 seconds, P = 0.0191 and P = 0.0066, respectively), and there was a shorter duration in the HLB group compared to the HB group (24.9 ± 5.8 vs. 33.1 ± 9.3 seconds, P = 0.0383) (Figure 2B).

After ACh administration, the maximum percentage increase in the MSA inner diameter was significantly lower in the HS group than in the HB and HLS groups (17.27 ± 5.41 vs. 37.79 ± 21.18 and 60.43 ± 13.86%, P = 0.0389 and P < 0.0001, respectively), was significantly higher in the HLB group (68.87 ± 17.70%) than in the HB group (P = 0.0045) and was significantly lower in the HB group than in the HLS group (P = 0.0390) (Figure 2C). The duration of vasodilatation was significantly longer in the HB group than in the HS group (50.2 ± 19.9 vs. 24.2 ± 10.4 seconds, P = 0.0119) (Figure 2D and Table S2).

### The effects of NE and ACh on blood perfusion of the MSAs

**Figure 3:**
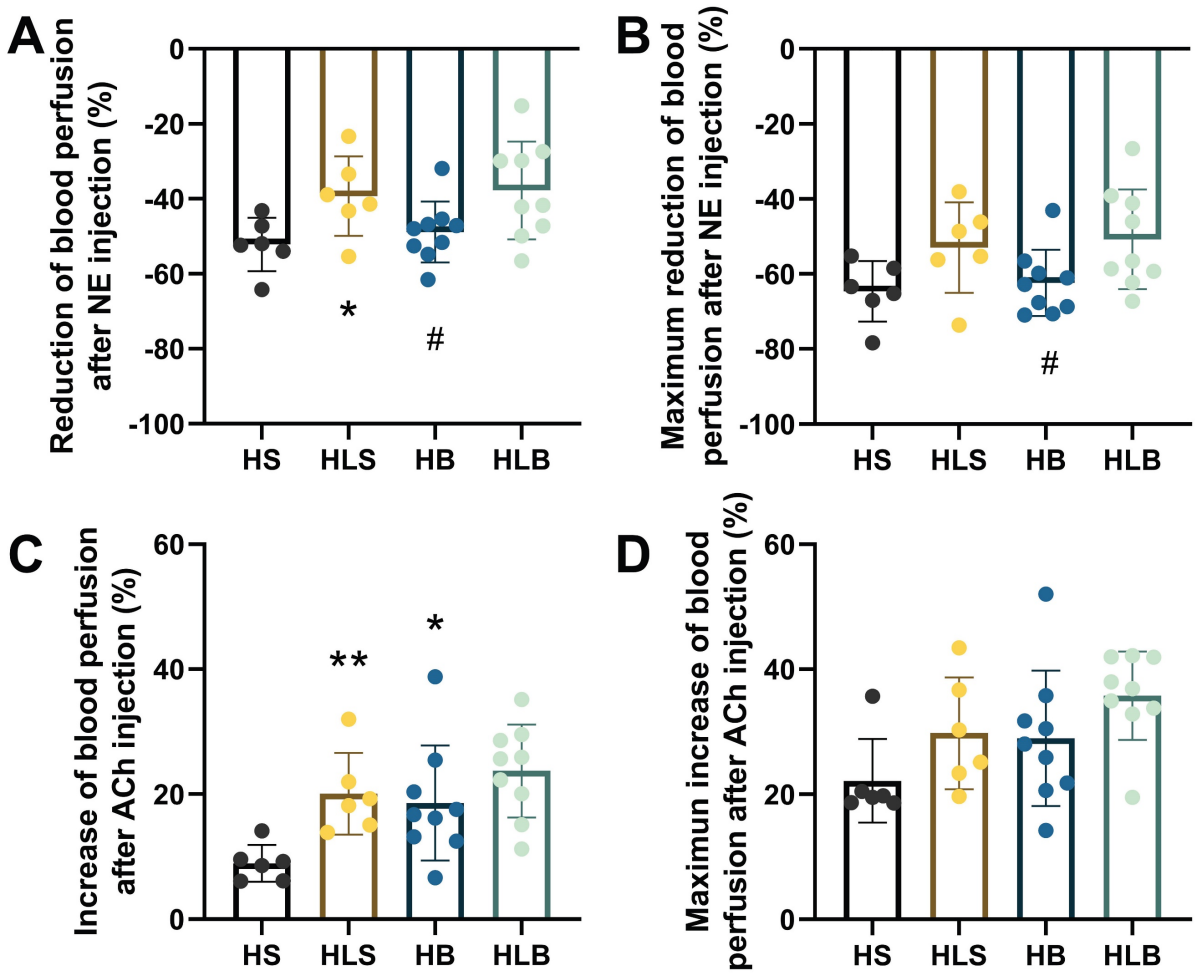
Blood perfusion of the MSAs was recorded by full-field laser perfusion imaging. The MSAs of the proximal small intestine were recorded continuously for at least 10 minutes before and after injection of NE or ACh. (A) The average percentage changes in blood perfusion after NE injection. (B) The maximum percentage changes in blood perfusion after NE injection. (C) The average percentage increase in blood perfusion induced by ACh. (D) The maximum percentage increase in blood perfusion induced by ACh. The percentage changes within the MSAs were calculated as the average change in blood perfusion within a thirty-second interval after drug injection divided by baseline blood perfusion (i.e., thirty seconds of blood perfusion before NE/ACh administration). The maximum percentage change was calculated as the maximum blood perfusion induced by ACh (or minimum blood perfusion induced by NA) divided by the baseline blood perfusion. The measurements of three MSA segments were averaged. HS: the group with high salt intake for 12 weeks, n=8; HLS: the group with high salt for the first 6 weeks, then low salt intake for the next 6 weeks, n=8; HB: the group with high salt intake plus Benazepril administered intragastrically for 12 weeks, n=9; HLB: the group with high salt for the first 6 weeks, then low salt intake for the next 6 weeks, and with Benazepril administration throughout 12 weeks, n=9. MSA: mesenteric small artery; NE: norepinephrine; ACh: acetylcholine. *: compared with the HS group, P<0.05; **: compared with the HS group, P<0.01; #: compared with the HLB group, P<0.05.

The baseline MSA blood perfusion did not significantly differ among the 4 groups before NE and ACh administration. After NE injection, the average percentage reduction in the MSA blood perfusion in the HLS group was significantly less than that in the HS group, and that in the HB group was also lower than that in the HLB group (HLS vs. HS: -39.25 ± 10.65 vs. -52.15 ± 7.13%, P = 0.0334; HLB vs. HB: -37.74 ± 13.07 vs. -48.83 ± 8.10, P = 0.0459, respectively); there was no difference between the HB and HLS groups (P = 0.0689) (Figure 3A). The maximum percentage reduction in blood perfusion after NE injection was significantly greater in the HB group than that in the HLB group (-62.35 ± 8.85% vs. -50.76 ± 13.28, P=0.0447), and there was no difference between the HB and HLS groups (P=0.1056) (Figure 3B).

After ACh injection, ACh significantly increased MSA blood perfusion in the HB and HLS groups compared with the HS group (18.59 ± 9.20 and 20.05 ± 6.52 vs. 8.94 ± 2.96%, P = 0.0288 and P = 0.0035, respectively), however, HB and HLS did not differ (P = 0.7431) (Figure 3C). MSA blood perfusion, especially the maximum percentage change, was not significantly different among the 4 groups induced by ACh (Figure 3D).

### Histological results

**Figure 4:**
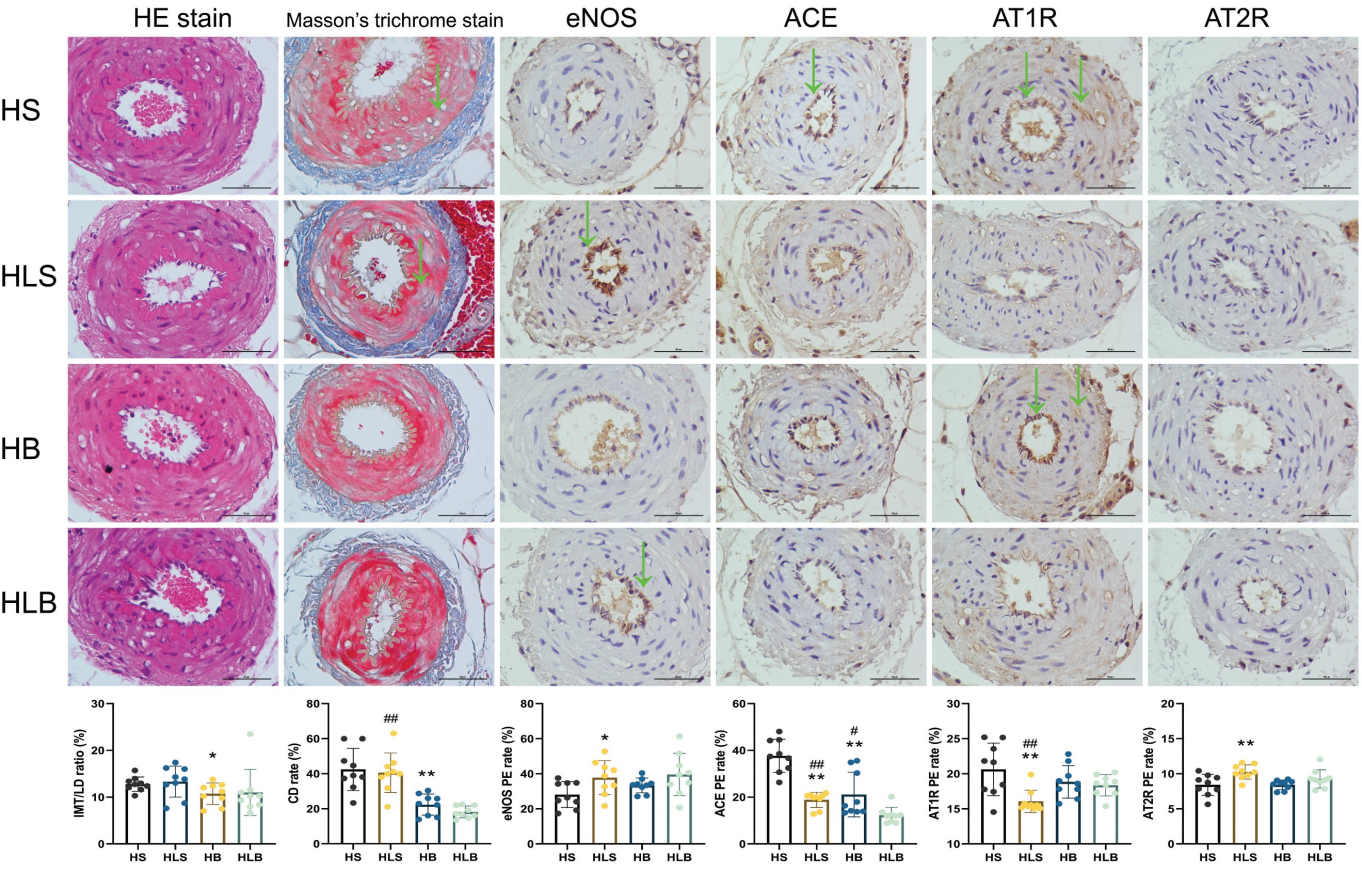
Histological and immunohistochemical changes in the MSAs of Dahl-SS rats. The sectioned MSAs were stained with hematoxylin and eosin (HE), Masson's trichrome and immunohistochemistry (original magnification × 400, scale bar: 50 μm). For IHC, nuclei appeared brown in positive expression of MSA by immunohistochemistry assay. HS: the group with high salt intake for 12 weeks; HLS: the group with high salt for the first 6 weeks, then low salt intake for the next 6 weeks; HB: the group with high salt intake plus Benazepril administered intragastrically for 12 weeks; HLB: the group with high salt for the first 6 weeks, then low salt intake for the next 6 weeks, and with Benazepril administration throughout 12 weeks. MSA: mesenteric small artery; HE: hematoxylin and eosin; IHC: immunohistochemical staining; eNOS: endothelial nitric oxide synthase; ACE: angiotensin-converting enzyme; AT1R: angiotensin II type 1 receptor; AT2R: angiotensin II type 2 receptor. IMT/LD ratio: intima-media thickness/internal lumen diameter ratio; CD rate: collagen deposition rate. eNOS PE rate: positive expression rate of endothelial nitric oxide synthase; ACE PE rate: positive expression rate of angiotensin converting enzyme; AT1R PE rate: positive expression rate of angiotensin II type 1 receptor; AT2R PE rate: positive expression rate of angiotensin II type 2 receptor. *: compared with the HS group, P<0.05; **: compared with the HS group, P<0.01; #: compared with the HLB group, P<0.05; ##: compared with the HLB group, P<0.01; @: compared with the HB group, P<0.05; @@: compared with the HB group, P<0.01.

Figure 4 shows the histopathological changes in MSAs. After the 12-week intervention, according to the pathological sections, there was no significant difference in smooth muscle proliferation, intima-media thickness and internal lumen diameter among the 4 groups. In the HS group, collagen deposition rates in the intima-media of MSAs were significantly higher than in the HB group (P = 0.0004), according to Masson trichrome staining. Similar phenomenon was shown in HLS group than that in the HLB group (P < 0.0001).

A significant difference existed between the HLS and HS groups in terms of eNOS expression in the MSAs by immunohistochemistry (P = 0.0308), and there was no difference between the HB and HLS groups (P = 0.2295). The ACE expression in the MSAs was significantly lower in the HB and HLS groups than in the HS group (P = 0.0007 and P < 0.0001, respectively) and higher than that in the HLB group (P = 0.0182 and P = 0.0007, respectively), but there was no difference between the HB and HLS groups (P = 0.5063). The AT1R expression in the MSAs were significantly lower in the HLS group than in the HS, HLB, and HB groups (P = 0.0041, P = 0.0064, and P = 0.0091, respectively). Compared to HS and HB, the HLS group showed significantly higher levels of AT2R in the MSAs (P = 0.0079 and P = 0.0004, respectively) (Figure 4 and Table S3).

## Discussion

In this work, as shown in Dahl-SS rats, salt restriction improves resistance arteries' response to vasoactive agents in real-time when compared with benazepril. Six weeks of salt restriction had a comparable BP-lowering effect to benazepril in Dahl-SS rats. Both switching from high to low salt intake and ACE inhibitor treatment favorably affected NE-induced vasoconstriction and ACh-induced vasodilatation in the MSAs of rats, and the effect intensity of salt restriction was comparable to that of ACE inhibitors. The immunohistochemistry study showed that salt restriction was associated with an inhibition of the local RAS and an improvement in eNOS expression.

The hypertension and cardiovascular disease guidelines 1, 4, 21, 22 strongly recommend hypertensive patients, especially those with high salt intake and salt-sensitive individuals, to reduce salt intake. Most studies compared long-term intervention of high salt to low-salt diet, lacking a process of switching salt intake, which is a common lifestyle change of hypertensive people in real clinical world. Major gaps remain regarding the relationship of salt intake with human physiology and health. Although previous studies 23-25 have shown that high salt intake negatively impacts the response of resistant arteries to vasoactive agents, there was a lack of research focusing on the improvement effects of switching from a high- to low-salt diet *in vivo*. In a human study 26, found that salt reduction improved endothelium-dependent vasodilation 26 but did not improve arterial stiffness 27 and showed no impact on central and peripheral hemodynamics during exercise 28. Perhaps it is quite impractical in human studies to design sophisticated procedures to observe the effects of salt restriction on vascular function. Therefore, our experiments used living animals through a sophisticated design to reveal the real-time effects of salt restriction on the function of MSAs, a phenomenon different from that of central and peripheral arteries and different from isolated arteries.

An animal study 29 showed that salt restriction can decrease arterial stiffness. The current study shows that salt restriction in salt-sensitive animals can not only reduce BP but also achieve significant improvement in vascular function in MSAs. As the purpose of lowering BP is to protect target organs, the improvement of vascular function may bring more benefits to individuals than the reduction of BP per se 30. In this study, salt restriction for 6 weeks in the high-salt diet individuals did not completely reverse high BP and vascular dysfunction to the initial state. As this study showed that low salt elevated angiotensin II and aldosterone levels, the RAS was also activated by low salt in Sprague-Dawley rats 31. Moreover, a previous study 32 showed that ong-term low salt diet-induced hypertension may be caused by activation of the RAS. Therefore, the effect of local RAS activated by a high-salt diet for a long time may not be completely eliminated 33, and the change to a low-salt diet activated circulating RAS, which may lead to some of the changes developed during salt loading that could not be reversed.

The function and structure of arteries can be improved by ACE inhibitors according to a large number of previous studies 34-36. ACE inhibitors also reverse vascular dysfunction and hyperconstriction of peripheral arteries 37. In this *in vivo* study, an ACE inhibitor improved RAS activation, both vasoconstriction and vasodilatation, in Dahl-SS rats, but the improvement in vasoconstriction was more obvious. Vasoconstriction is mainly driven by vascular smooth muscle, while ACE inhibition results in improved vascular function by reducing vascular smooth muscle activation and vessel wall remodeling 38. The angiotensin receptors AT1R and AT2R play opposite roles in regulating sodium excretion, and the increased AT1R activity reduces sodium excretion in the proximal tubule and distal tubule and plays a stronger role 39. Interestingly, in pathological conditions of sodium depletion, the expression of AT2R was enhanced compared with that in healthy tissue 40, 41. In a study 42, chronic treatment with an AT2R agonist prevented salt-sensitive hypertension. However, ACE inhibitor treatment reduced the activation of both AT1 and AT2 receptors to improve sodium and fluid retention. The current study shows that switching from high to low salt intake almost achieved comparable effects on the local RAS as using ACE inhibitor in high salt intake animals after 6-week intervention, meanwhile, salt restriction slightly increased the AT2R expression without ACE inhibitor. Both excessively low- 43 and high-salt diets cause local RAS activation. Our study showed that moderately low salt levels can improve RAS activation, including downregulating the expression of ACE and AT1R and upregulating the expression of AT2R.

A dysfunctional activated circulatory RAS maintains high blood pressure 44, while activated tissue RAS damages and malfunctions target organs 45. The chronic activation of RAS can participate in structural and functional alteration of the arterial wall 46, 47. In the current study, salt restriction was associated with an upregulation of eNOS expression and an inhibition of local RAS activity, including downregulation of ACE and AT1R in the MSAs. These results were similar to those of the ACE inhibitors, and may be related to the reversal of unfavorable response of the MSAs to vasoactive agents. Moreover, ACE inhibitor treatment plus salt restriction could inhibit local RAS and promote eNOS expression synergistically in animals with salt-sensitive hypertension. The results showed that salt restriction was almost equivalent to the same inhibitory effect on the local RAS of ACE inhibitors, which may be a change in a short time. In a study 48 of high fructose-induced hypertension in Dahl-SS rats, an ACE inhibitor attenuated hypertension and renal damage after 12 weeks of treatment. According to the inhibition of local RAS based on salt restriction, ACE inhibitors may have a more obvious inhibition effect in the long-range that may be longer than 12 weeks, but further exploration is needed.

We attempted to elucidate the critical role of salt restriction and ACE inhibitors in salt-sensitive hypertension, but this study had some limitations. First, we have described a remarkable phenomenon with immunohistochemistry but lack further mechanistic exploration. Second, we have shown in animals that salt restriction was almost as effective as ACE inhibitors in MSAs of Dahl-SS rats, but the implication in clinical practice remains to be studied. Third, it is difficult to switch from a high- to low-salt diet in real clinical practice due to poor patient compliance. Therefore, we enthusiastically explored and emphasized the favorable impacts of salt restriction on vascular function. Fourth, salt restriction and ACE inhibitors did not return BP values to baseline but only prevented further increases in the 6^th^ to 12^th^ week period. It would have been interesting to have weekly BP measurements to better appreciate the BP trend. Finally, we are actually interested in the impact of different levels of salt intake on BP and vascular function; unfortunately, we only set two grades of salt intake in the present study.

In conclusion, we found that switching from high-salt to low-salt diets and ACE inhibitor treatment improved the responsiveness of resistant arteries to vasoactive agents synergistically. The expression of ACE, AT1R, and eNOS at the local level may aid these processes. Moreover, the improvements in BP and vascular function highlight the importance of salt restriction for hypertension and shed light on reducing salt-sensitive hypertension.

## Supplementary Material

Supplementary figure and tables, video legend.

Supplementary video 1.

## Figures and Tables

**Table 1:** Blood and urine analysis at the end of 12^th^ week.

	HS (n=8)	HLS (n=8)	HB (n=9)	HLB (n=9)
AT II (pg/ml)	135.75±8.86	170.37±12.34 **	154.36±31.52	178.80±47.61
ALD (pg/ml)	15.48±3.51	35.79±2.01 **, ##, @@	22.37±4.30 **	25.22±4.06
Cr (μmol/L)	31.76±8.09	27.35±1.68 #	26.50±4.83	24.68±3.14
K+ (mmol/L)	4.33±0.66	4.68±0.25	4.32±0.47	4.58±0.25
24 hU-Na (mmol/24h)	3.27±1.02	0.38±0.18 **, @@	2.21±1.53 ##	0.34±0.08
24 hU-Pro (mg/24h)	139.21±70.39	91.47±27.51 *, @	63.40±21.46 **	60.69±26.53

Notes: AT II: plasma angiotensin II; ALD: plasma aldosterone; Cr: serum creatinine; K+: serum potassium; 24 hU-Na: 24 hours urinary sodium; 24 hU-Pro: 24 hours urinary total protein. HS: high salt intake group; HB: high salt intake with Benazepril administered intragastrically; HLS: HS for the first 6 weeks, then low salt intake for the next 6 weeks; HLB: HLS with Benazepril administration. *: compared with HS group, P<0.05; **: compared with HS group, P<0.01; #: compared with HLB group, P<0.05; ##: compared with HLB group, P<0.01; @: compared with HB group, P<0.05; @@: compared with HB group, P<0.01.
