# Supplementary material for: Salt Restriction and Angiotensin-Converting Enzyme Inhibitor Improve the Responsiveness of the Small Artery in Salt-Sensitive Hypertension
Source: Int J Med Sci. 2023 Mar 5;20(5):572–80. doi: 10.7150/ijms.79741 (PMC10110468; doi:10.7150/ijms.79741)
Supplement: Supplementary file 1 — Supplementary figure and tables, video legend. [file ijmsv20p0572s1.pdf]

Table S1. The blood pressure and heart rate of Dahl-SS rats at baseline, the end of the 6-week and 12-week dietary intervention.

	**HS group**	**HLS group**	**HB group**	**HLB group**
**Baseline**				
SBP (mm Hg)	122.13±1.98	123.25±5.66	122.30±2.68	123.41±3.13
DBP (mm Hg)	104.29±2.64	106.96±3.15	106.37±3.42	104.22±1.94
MBP (mm Hg)	110.24±1.60	112.39±3.35	111.68±2.61	110.62±1.54
HR (beats per minute)	408.29±12.37	403.92±14.83	418.81±15.82	406.78±11.78
**At the end of 6th week**				
SBP (mm Hg)	162.04±9.35	159.17±6.85 @@, ##	143.52±3.85 **	145.11±9.76
DBP (mm Hg)	135.83±8.45	135.54±7.55 @@, #	118.78±11.12 **	120.93±13.50
MBP (mm Hg)	144.57±8.34	143.42±6.63 @	127.02±8.30 **	128.99±12.15
HR (beats per minute)	404.71±10.78	409.13±12.14 @, #	429.48±18.42 **	431.30±19.78
**At the end of 12th week**				
SBP (mm Hg)	182.04±9.18	160.67±10.51 **, #	155.37±3.49 **, #	145.00±11.53
DBP (mm Hg)	153.42±6.66	135.58±10.21 **, @, ##	126.30±6.45 **	118.78±11.85
MBP (mm Hg)	162.80±7.11	144.13±9.81 **, ##	136.82±5.50 **, #	128.04±11.35
HR (beats per minute)	435.25±18.44	421.71±18.71	419.19±18.69	409.04±28.19

Notes: SBP: systolic blood pressure; DBP: diastolic blood pressure; MBP: mean blood pressure; HR: heart rate; HS: high salt intake group; HB: high salt intake with Benazepril administered intragastrically; HLS: HS for the first 6 weeks, then low salt intake for the next 6 weeks; HLB: HLS with Benazepril administration. MSA: mesenteric small artery; NE: Norepinephrine; ACh: acetylcholine. *: compared with HS group, P<0.05; **: compared with HS group, P<0.01; #: compared with HLB group, P<0.05; ##: compared with HLB group, P<0.01; @: compared with HB group, P<0.05; @@: compared with HB group, P<0.01.

Table S2. The minimum/maximum diameters and maximum percentage change of MSA after NE or ACh injection.

	**HS group**	**HB group**	**HLS group**	**HLB group**
**After NE injection**				
Maximum percentage reduction of inner diameter (%)	50.48±11.34	42.25±14.39 #	34.13±10.27 *	25.03±12.08
Duration of vasoconstriction (second)	51.59±17.52	33.12±9.22 *, #	26.00±5.51 **	24.86±5.78
Time to maximum vasoconstriction (s)	31.40±19.20	27.41±8.41 ##	16.55±4.38 @	17.63±3.89
**After ACh injection**				
Maximum percentage change of inner diameter (%)	17.26±5.41	37.79±21.18 *, ##	60.43±13.86 **, @	68.88±18.70
Duration of vasodilatation (second)	24.23±10.37	50.21±19.89 *	37.05±11.26 *	43.96±11.38
Time to maximum vasodilatation (s)	24.86±8.64	36.71±16.64	30.45±7.17	27.98±6.94

Notes: The MSAs were record by a high-speed camera attached to the microscope with using GigaView Suite software. To clarify vasoconstrictive properties of the MSAs, the maximum percentage change in vasoconstriction was calculated as [(Dbn – Dn)/Dbn]×100%. Dbn was the baseline inner diameter of the artery before NE administration, and Dn was the most vasoconstricted inner diameter after NE injection. To clarify the vasodilated effect on the MSAs, the maximum percentage change in vasodilatation of the MSA was calculated as [(Da-Dba)/Dba]×100%. Dba was the baseline inner diameter before ACh administration, and Da was the maximum vasodilated inner diameter after ACh was given. MSA diameters were determined from images evaluated by Image-Pro Plus 6.0 software. HS: high salt intake group; HB: high salt intake with Benazepril administered intragastrically; HLS: HS for the first 6 weeks, then low salt intake for the next 6 weeks; HLB: HLS with Benazepril administration. MSA: mesenteric small artery; NE: Norepinephrine; ACh: acetylcholine. *: compared with HS group, P<0.05; **: compared with HS group, P<0.01; #: compared with HLB group, P<0.05; ##: compared with HLB group, P<0.01; @: compared with HB group, P<0.05.

Table S3. Morphological and immunohistochemical characteristics of MSA after 12-week diet intervention.

	HS (n=8)	HLS (n=8)	HB (n=9)	HLB (n=9)
Intima-media thickness (μm)	18.72±6.05	20.33±5.44	16.33±5.78	16.52±11.44
Internal lumen diameter (μm)	143.48±31.73	153.82±24.99	151.45±37.23	143.60±26.60
IMT/LD ratio (%)	12.79±1.52	13.32±3.31	10.73±2.32 *	11.00±4.94
CD rate (%)	42.43±12.10	40.56±11.24 ##, @@	22.33±6.04 **	18.20±3.39
eNOS PE rate (%)	28.12±7.43	37.74±9.67 *	33.36±4.19	39.59±12.10
ACE PE rate (%)	37.66±7.11	18.85±3.27 **, ##	21.14±9.55 **, #	12.24±3.43
AT1R PE rate (%)	20.62±3.76	16.07±1.59 **, ##, @@	18.85±2.32	18.36±1.51
AT2R PE rate (%)	8.43±1.50	10.26±1.01 **, @@	8.43±0.67	9.27±1.31

Notes: IMT/LD ratio: Intima-media thickness/ Internal lumen diameter ratio; CD rate: Collagen deposition rate. eNOS PE rate: Positive expression rate of endothelial nitric oxide synthase; ACE PE rate: Positive expression rate of angiotensin converting enzyme; AT1R PE rate: Positive expression rate of angiotensin II type 1 receptor; AT2R PE rate: Positive expression rate of angiotensin II type 2 receptor. Antibodies dilution ratio: eNOS, 1:500; ACE, 1:200; AT1R, 1:100; AT2R, 1:500. HS: high salt intake group; HB: high salt intake with Benazepril administered intragastrically; HLS: HS for the first 6 weeks, then low salt intake for the next 6 weeks; HLB: HLS with Benazepril administration. *: compared with HS group, $P<0.05$; **: compared with HS group, $P<0.01$; #: compared with HLB group, $P<0.05$; ##: compared with HLB group, $P<0.01$; @: compared with HB group, $P<0.05$; @@: compared with HB group, $P<0.01$.

Figure S1. The maximum diameter and blood perfusion change in the MSAs after NE and ACh injection, respectively, as recorded by a high-speed camera attached to a microscope (A) and LPI (B). The baseline inner and outer diameters of the MSAs were recorded before injection (A1). After NE and Ach injection respectively, the maximum vasoconstriction (A2) and vasodilatation (A3) of the MSAs were measured. The baseline blood perfusion of the MSAs before NE and ACh injection. Comparing with the baseline the perfusion of MSAs (B1), those were significantly lower after NE injection (B2). After ACh injection (B3), the blood perfusion of MSAs were more significant than baseline. MSA: mesenteric small artery; NE: Norepinephrine; ACh: acetylcholine.

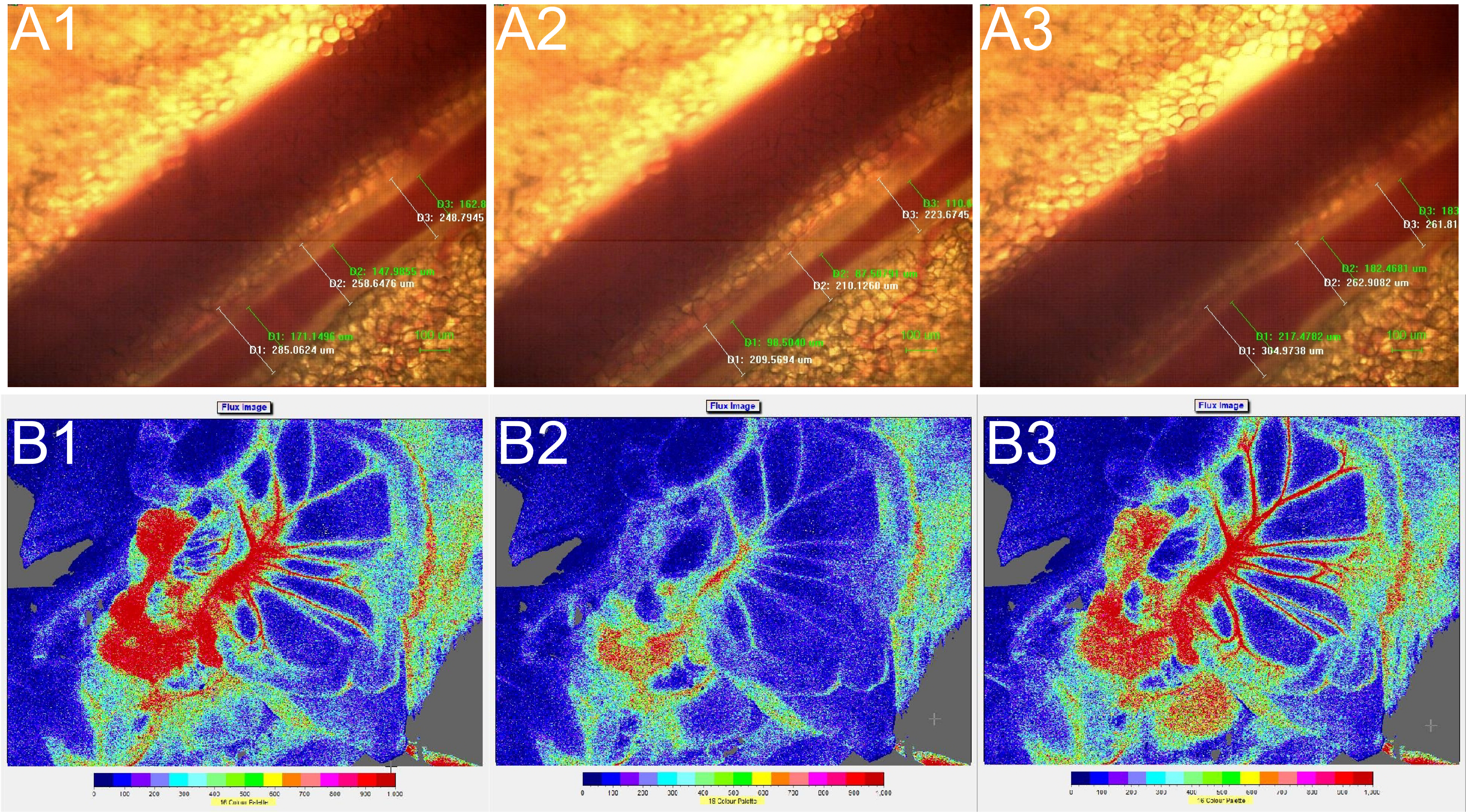
A1
D3: 162.8
D3: 248.7945
D2: 147.9855 um
D2: 258.6476 um
D1: 171.1496 um
D1: 285.0624 um
100 um
A2
D3: 223.6745
D2: 210.1260 um
D1: 98.5040 um
D1: 209.5694 um
100 um
A3
D3: 183
D3: 261.81
D2: 182.4681 um
D2: 262.9082 um
D1: 217.4782 um
D1: 304.9738 um
100 um
Flux Image
B1
Flux Image
B2
Flux Image
B3

Video S1. The video shows the NE-induced vasoconstriction and ACh-induced vasodilatation of MSA, as recorded by a high-speed camera attached to a microscope and processed by GigaView Suite software.
